# Actions and Consequences of Insulin in the Striatum

**DOI:** 10.3390/biom13030518

**Published:** 2023-03-11

**Authors:** Jyoti C. Patel, Kenneth D. Carr, Margaret E. Rice

**Affiliations:** 1 Department of Neurosurgery, New York University Grossman School of Medicine, New York, NY 10016, USA; 2 Department of Psychiatry, New York University Grossman School of Medicine, New York, NY 10016, USA; 3 Department of Biochemistry and Molecular Pharmacology, New York University Grossman School of Medicine, New York, NY 10016, USA; 4 Department of Neuroscience and Physiology, New York University Grossman School of Medicine, New York, NY 10016, USA

**Keywords:** dopamine, acetylcholine, obesity, flavor–nutrient learning, reward, mood

## Abstract

Insulin crosses the blood–brain barrier to enter the brain from the periphery. In the brain, insulin has well-established actions in the hypothalamus, as well as at the level of mesolimbic dopamine neurons in the midbrain. Notably, insulin also acts in the striatum, which shows abundant expression of insulin receptors (InsRs) throughout. These receptors are found on interneurons and striatal projections neurons, as well as on glial cells and dopamine axons. A striking functional consequence of insulin elevation in the striatum is promoting an increase in stimulated dopamine release. This boosting of dopamine release involves InsRs on cholinergic interneurons, and requires activation of nicotinic acetylcholine receptors on dopamine axons. Opposing this dopamine-enhancing effect, insulin also increases dopamine uptake through the action of insulin at InsRs on dopamine axons. Insulin acts on other striatal cells as well, including striatal projection neurons and astrocytes that also influence dopaminergic transmission and striatal function. Linking these cellular findings to behavior, striatal insulin signaling is required for the development of flavor–nutrient learning, implicating insulin as a reward signal in the brain. In this review, we discuss these and other actions of insulin in the striatum, including how they are influenced by diet and other physiological states.

## 1. Introduction

Since the discovery of insulin 100 years ago, a primary focus of insulin research has been to understand how this metabolic hormone regulates glucose homeostasis. The body of evidence gained has been fundamental for developing treatment strategies for conditions involving insulin resistance, including obesity-sensitive type 2 diabetes [1]. However, the actions of insulin in the body are not just metabolic in nature, and are not restricted to the periphery. Pancreatic insulin can enter the brain slowly at the level of the choroid plexus, which produces cerebrospinal fluid (CSF) in the ventricles of the brain. Ventricular CSF is contiguous with brain interstitial fluid, allowing a gradual increase in brain insulin levels following insulin release in the periphery. A more direct and rapid source for brain entry, however, is by trans-endothelial transport at the level of brain microvasculature, i.e., by crossing the blood–brain barrier (BBB) [2,3,4,5]. In addition, it has been suggested that insulin may be produced and released at the choroid plexus [6], and even by some neurons [7,8] and astrocytes [9] within the brain, providing an even more local and rapid source of insulin.

Physiological concentrations of insulin in CSF and the brain are estimated to reach 10 to 30 nM after food intake [2,10,11,12,13]. Within the brain, mRNA transcripts and protein for insulin receptors (InsRs) are distributed widely with high levels in the olfactory bulb, hypothalamus, hippocampus, cortex, cerebellum, midbrain and striatum [14,15,16,17,18,19,20]. Thus, insulin has the potential to impact multiple brain regions and their functions through InsR signaling. Insulin also acts at insulin-like growth factor 1 receptors (IGF-1Rs), albeit at higher concentrations than those required to activate InsRs [17,21], as well as to hybrid receptors containing InsR subunits with IGF-1R subunits [22]. Both receptor types, however, use similar downstream signaling machinery [23,24,25,26]. Consequently, discerning actions of insulin in the brain through InsRs vs. IGF-1Rs is vital for understanding insulin’s effect on brain function and for specific targeting of appropriate receptor-mediated signaling components to combat brain dysfunction.

The most studied function of insulin in the brain is its role as a satiation signal acting in the hypothalamus to limit food intake during a meal, as well as to indicate satiety between meals [27,28]. However, this is far from the only role of insulin in the brain, with increasing evidence for its influence on diverse brain functions, ranging from non-homeostatic hedonic feeding and food choices to cognition and regulation of emotional tone [29,30,31,32,33,34,35,36]. Importantly, development of insulin resistance in the periphery also extends to the brain, with mechanisms and consequences of dysregulated insulin signaling in specific regions largely under explored.

In recent years, much interest has been focused on the actions of insulin on mesolimbic dopamine neurons in the midbrain that drive motivated behavior. Selective activation of InsRs on ventral tegmental area (VTA) dopamine neurons opposes somatodendritic dopamine release by increasing dopamine uptake [37]. Moreover, insulin in the VTA impairs glutamatergic transmission onto dopamine neurons [38] and thereby decreases dopamine release from VTA axon projections in the ventral striatum [39], which leads to decreased feeding behavior [37,38]. The details of the actions of insulin in the VTA have been recently reviewed [30,34]. However, insulin also has distinct actions in the striatum that compete with those at the level of dopamine neurons in the VTA.

Here we review the actions of insulin in the striatum, a forebrain region crucial for movement, mood, and motivated behavior, functions that are strongly influenced by its dense dopaminergic innervation that arises from midbrain dopamine neurons in the substantia nigra pars compacta (SNc) and VTA. This dopaminergic input regulates the activity of striatal projection neurons (SPNs), which provide the sole neuronal output from the striatum. We describe how insulin influences striatal dopamine axons through activation of sparse but influential cholinergic interneurons (ChIs) to promote dopamine release, and also acts directly on dopamine axons to enhance dopamine uptake. Complementary behavioral studies show that the actions of endogenous insulin in a subregion of the ventral striatum, the nucleus accumbens (NAc) shell, regulates the development of nutrient-based flavor preference, a dopamine-dependent process. Moreover, the influence of insulin in food choice is oppositely affected by food restriction or the availability of high energy/obesogenic diets. We also consider the actions of insulin on SPNs and on striatal astrocytes, as well as the broader implications of the actions of insulin in the striatum on dopamine release regulation and its possible role in anxiety and depressive-like behaviors.

## 2. Insulin Promotes Striatal Dopamine Release through ChIs and nAChRs

Striatal dopamine release is governed by the activity of midbrain dopamine neurons, but can also be triggered from within the striatum by activation of nicotinic acetylcholine receptors (nAChRs) that are enriched on dopamine axons [40,41,42,43]. The source of acetylcholine (ACh) for this process are ChIs, which make up only a small percentage of the neuronal population of the striatum, yet exert a powerful influence on dopamine release and striatal function through extensive axonal processes that enable non-synaptic, as well as synaptic-like, transmission with dopamine axons [43].

**Figure 1:**
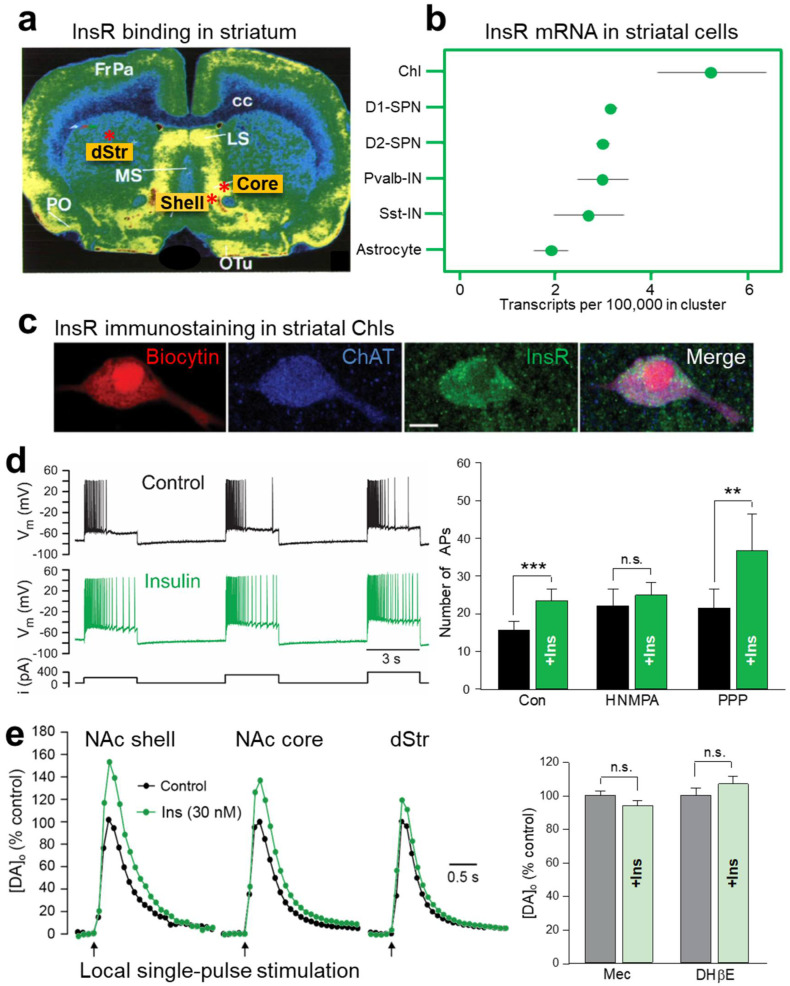
InsR localization in striatum and insulin-dependent regulation of striatal dopamine release via ChIs, InsRs and nAChRs. (**a**) Autoradiographic image of insulin receptor (InsR) binding sites throughout the rat striatum, including in the dorsal striatum (dStr), nucleus accumbens (NAc) core and shell subregions marked by red asterisks; modified with permission from [16]. Abbreviations: cc, corpus callosum; FrPa, frontal parietal cortex; LS, lateral septum; MS, medial septum; PO, preoptic area; oTu, olfactory tubercle. (**b**) Comparison of relative mRNA levels for InsRs in striatal cells shows high abundance in cholinergic interneurons (ChIs) versus D1 or D2 spiny projection neurons (SPNs), parvalbumin positive interneurons (Pvalb-IN), somatostatin positive interneurons (Sst-IN) or astrocytes; RNA-seq data was obtained using DropViz. (**c**) Immunohistochemical staining of InsRs in a biocytin-filled, ChAT-positive ChI; scale = 10 μm; images from [19]. (**d**) Left: Response of current-clamped rat ChI to depolarizing current pulses; insulin (30 nM) increases ChI excitability by attenuating action potential (AP) accommodation. Right: Insulin-induced increase in ChI excitability is prevented by an InsR antagonist, HNMPA, but not by an IGF-1R antagonist, PPP; from [19]. (**e**) Left: Insulin (30 nM) enhances single-pulse evoked increases in extracellular dopamine concentration ([DA]_o_) in dStr but has a greater effect in NAc core and NAc shell subregions of rat striatal slices. Right: Insulin evoked increases in [DA]_o_ are prevented by nAChR antagonists, mecamylamine (mec) or DHβE; from [19]. For d and e, n.s. is not significant, ** *p* < 0.01, *** *p* < 0.001.

Early studies using autoradiography show abundant InsR binding sites throughout the striatum (Figure 1a) [15,16,17] but lack the resolution to discern which striatal cells express these InsRs. Analysis of RNA-seq data in DropViz (http://dropviz.org, accessed on 16 September 2022) [44], shows the highest levels of mRNA for InsRs in ChIs compared to other striatal cells including SPNs (Figure 1b). Consistent with abundant mRNA in ChIs, immunohistochemical examination of InsRs in rat striatum indicates dense labeling for InsR proteins in ChIs, identified by co-immunostaining for the primary enzyme for ACh synthesis, choline acetyltransferase (ChAT) (Figure 1c) [19]. Thus, ChIs provide a significant target by which insulin could influence dopamine transmission.

### 2.1. Insulin Increases the Excitability of Striatal ChIs

Electrophysiological recordings in ex vivo striatal slices have demonstrated functional consequences of InsR activation in ChIs. The method used is whole-cell recording, in which a micropipette containing a solution resembling the cytoplasm forms a continuum with an individual cell, enabling the electrical activity of the cell to be monitored. In experiments using current-clamp recording in which the current across a cell membrane is controlled and membrane potential is monitored, injecting positive current to depolarize a recorded neuron causes an initial burst of action potentials (APs) that shows a progressive decrease in frequency with continued depolarization. This is referred to as AP accommodation [45]. Addition of a physiological concentration of insulin (30 nM) to the artificial cerebrospinal fluid (aCSF) that superfuses the slice increases ChI excitability, seen as an increase in AP number during prolonged depolarization, i.e., attenuated AP accommodation (Figure 1d) [19]. This increase in excitability is InsR dependent, as the effect of insulin is prevented by an InsR inhibitor, HNMPA (hydroxy-2-naphthalenylmethylphosphonic acid), whereas blocking IGF-1Rs with PPP (picropodophyllin) has no effect (Figure 1d) [19]. The mechanism by which insulin increases ChI excitability is unresolved, but is likely to involve one or more ion channels governing the membrane dynamics of these neurons [45,46].

### 2.2. Insulin Boosts Dopamine Release via InsRs, and Requires PI3K and nAChRs

The action of insulin on ChI excitability is sufficient to have a profound impact on axonal dopamine release in striatal slices as monitored using fast-scan cyclic voltammetry (FSCV). FSCV is an electrochemical method used to monitor the profile of evoked increases in extracellular dopamine concentration ([DA]_o_) by detecting the current generated during the oxidation of dopamine at the surface of a carbon fiber microelectrode when a triangular voltage waveform is applied. The voltage waveform is typically applied every 100 ms and the carbon fiber microelectrode is typically 5 to 10 μm in diameter thereby providing a dopamine release profile with high temporal (subsecond) and high spatial (few micrometer) resolution [47,48]. Previous studies using FSCV show that up to 70% of dopamine release evoked by a single electrical pulse in striatal slices can be attributed to ChI-induced ACh release and consequent activation of nAChRs on dopamine axons [49,50,51,52]. Strikingly, application of low nM insulin via the aCSF amplifies single pulse-evoked [DA]_o_ further, with a greater enhancement in the reward-related NAc core and shell regions of the ventral striatum than in the motor-related dorsal striatum (dStr) (Figure 1e). This regional heterogeneity in the response to insulin mirrors the regional density of InsRs in the striatum (Figure 1a) [15,16,17]. Showing that the influence of insulin on dopamine release involves dynamic regulation of the exocytotic process, tissue dopamine content of striatal slices is unaltered when exposed to 30 nM insulin, eliminating potential contributions from changes in dopamine synthesis or storage [19].

Like the changes seen in ChI excitability, amplification of evoked [DA]_o_ by insulin is mediated by InsRs rather than IGF-1Rs; insulin-induced increases in dopamine release are prevented by HNMPA, but not by PPP [19]. Interestingly, a higher concentration of insulin, 100 nM, does not produce a significant enhancement of evoked dopamine release which could reflect competing activation of IGF-1Rs, as seen in SPNs (see Section 5), although this has not been investigated.

**Figure 2:**
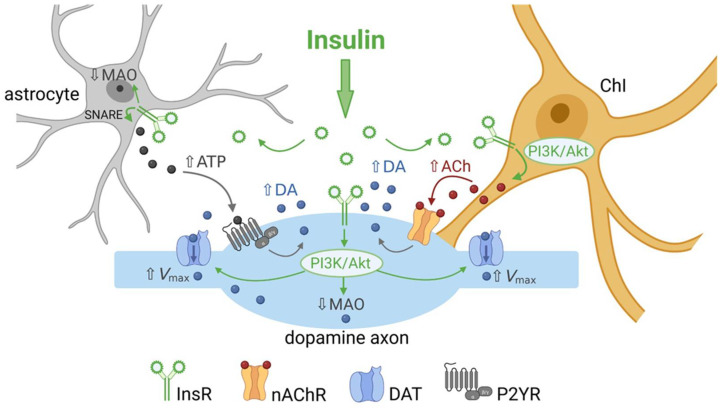
Summary of the multiple ways by which insulin can regulate striatal dopamine (DA) transmission. Activation of insulin receptors (InsRs) on cholinergic interneurons (ChIs) increases ChI excitability, which increases acetylcholine (ACh) release and activation of nicotinic ACh receptors (nAChRs) on dopamine axons to boost dopamine release and increase extracellular DA concentration ([DA]_o_). Activation of InsRs on dopamine axons increases dopamine transporter (DAT) surface expression via the PI3K/Akt pathway to increase the maximum velocity (*V*_max_) of dopamine uptake, thereby lowering [DA]_o_. Activation of InsRs on astrocytes increases exocytotic ATP release, which activates purinergic P2Y receptors (P2YR) on dopamine axons and enhances dopamine release. Insulin also decreases monoamine oxidase (MAO) activity in dopamine axons and glial cells, thereby decreasing dopamine metabolism and prolonging the time course of dopamine actions.

InsRs are members of the receptor tyrosine kinase superfamily which activate two major signaling pathways: the phosphatidylinositol 3-kinase (PI3K) pathway and the mitogen-activated protein kinase (MAPK) pathway [53]. Insulin-induced changes in evoked [DA]_o_ are prevented in the presence of a PI3K inhibitor, LY29002 in all striatal subregions, revealing the involvement of known downstream signaling mechanisms for insulin activation of InsRs in this process [19]. Demonstrating a pivotal ACh-DA interaction, the effect of insulin on evoked [DA]_o_ is prevented by mecamylamine, a non-selective nAChR antagonist, as well as by DHβE, a selective antagonist of β2 subunit containing (β2*) nAChRs (Figure 1e) [19]. Insulin amplifies evoked striatal [DA]_o_ in slices from mice, as well as from rats; however, the effect of insulin is absent throughout the striatum of mice with genetic deletion of *ChAT* (*ChAT* KO) [19]. Together these observations provide a model by which insulin activation of InsRs on ChIs induces enhancement of ChI activity which then boosts ACh-mediated dopamine release via nAChRs on dopamine axons (Figure 2).

## 3. Insulin Enhances Striatal Dopamine Uptake via DAT on Dopamine Axons

The dopamine transporter (DAT) is expressed exclusively in dopamine neurons in both the midbrain cell body region and forebrain dopamine axons in the striatum [54,55,56] and is crucial for curtailing the amplitude and duration of dopamine transients, as well as maintaining homeostatic levels of [DA]_o_ through transporter-mediated uptake [57,58]. Thus, [DA]_o_ at a given location and time reflects a balance between the opposing processes of release and uptake [51,52]. In addition, DAT-dependent dopamine uptake provides a major source of dopamine for vesicular uptake and storage for subsequent release [59].

In addition to the localization of InsRs in striatal cells, immunohistochemical analysis of striatal InsRs show expression on dopamine axons, identified by the presence of tyrosine hydroxylase, the primary enzyme required for dopamine synthesis [19]. Downstream effects of InsR activation include the regulation of dopamine uptake. Insulin increases expression of mRNA for DAT in midbrain dopamine neurons [60], which could theoretically increase total DAT protein levels. However, the most profound effect that insulin has on the DAT is its ability to redistribute DAT localization within a dopamine neuron. Although an integral part of the plasma membrane, DAT surface expression is dynamically regulated by endocytic trafficking mediated by a variety of intracellular signaling pathways, including PKC, which promotes DAT internalization, and the PI3K-Akt signaling pathway which has the opposite effect [61,62,63]. Given that both InsRs and IGF-1Rs belong to the receptor tyrosine kinase superfamily that couple to PI3K via phosphorylation of InsR substrates [23,24,25,26], insulin can influence DAT surface expression and function via these pathways. In striatum, it has been shown that in addition to enhancing dopamine release (see Section 2), insulin promotes DAT trafficking to the plasma membrane through PI3K-Akt signaling, thereby increasing overall DAT activity and dopamine uptake [61,62,64,65,66]. Dopamine uptake in the striatum is governed by Michaelis–Menten kinetics [67] and has been assessed using several different methods and experimental preparations. Although the values for the uptake kinetic parameters obtained are not directly comparable across studies [68], the general consensus is that insulin increases the maximal uptake velocity, *V*_max_, for dopamine with little or no effect on *K*_m_, which is inversely related to the affinity of the DAT for dopamine [61,64,65,66,69]. Moreover, although dopamine uptake is less efficient in the ventral striatum than in the dorsal striatum [70,71,72], enhanced dopamine uptake by insulin is seen throughout the striatal complex and in both rats and mice [64,65,66,69].

Confirming activation of the PI3K-Akt pathway, insulin-induced increases in striatal DAT activity are prevented by PI3K inhibitors [61,64,66,69]. In addition, it has been shown that inhibiting PI3K alone can decrease *V*_max_ for dopamine uptake into synaptosomes (isolated membrane bound axonal segments containing vesicles and mitochondria) implying that this pathway tonically regulates DAT activity [61]. However, in studies using striatal slices, a PI3K inhibitor, LY29002, prevents insulin-mediated increases in dopamine uptake without altering tonic regulation of DAT function [66]. Notably, although PI3K can be activated by insulin acting at either InsRs or IGF-1Rs [53], blocking InsRs with HNMPA occludes the effect of 30 nM insulin on dopamine uptake, whereas blocking IGF-1Rs with PPP does not [66]. Thus, insulin-induced enhancement of dopamine uptake in the striatum is mediated by InsRs and the PI3K pathway in dopamine axons (Figure 2).

The enhancing effect of insulin on dopamine uptake would be expected to decrease evoked [DA]_o_, as seen in the VTA [37]. However, as described in Section 2, studies using FSCV detect elevated increases in [DA]_o_ in the presence of insulin [19,69], which reflects the dominant effect of insulin on increasing ACh-induced dopamine release. When striatal ACh is absent, as in *ChAT*-KO mice [50], insulin’s action on dopamine uptake prevails, revealing a net decrease in evoked [DA]_o_ with insulin application [66]. Given that DAT is a mediator of the short-term plasticity of dopamine release [73], insulin’s actions on DAT expression and dopamine uptake will have implications for overall dopamine transmission, independent of the influence of ChIs on dopamine release.

## 4. Insulin Inhibits Dopamine Metabolism

In addition to enhancing dopamine release through nAChRs and increasing dopamine uptake through the DAT, there is evidence that insulin can alter dopamine metabolism [31,74,75]. Key metabolizing enzymes for dopamine are monoamine oxidase enzymes (MAOs), which may be located in dopamine axons or in astrocytes (see Figure 2). In rodents, MAO mainly catalyzes the formation of the dopamine metabolite dihydroxyphenylacetic acid (DOPAC). Samples of striatal extracellular fluid taken using microdialysis in freely moving rats show a profound and long-lasting decrease in DOPAC levels following a peripheral insulin injection [74]. Moreover, mice with brain-wide deletion of InsRs have increased MAO expression in the striatum [75]. Consequently, total area under the curve for single-pulse evoked [DA]_o_ in the dStr and NAc is lower in striatal slices from mice lacking brain InsRs than from controls. Although peak amplitude was the same between genotypes, the overall duration of the release response was shortened [75]. This suggests another route by which endogenous insulin could enhance [DA]_o_, through inhibition of dopamine metabolism via MAO suppression (Figure 2).

## 5. Insulin Bidirectionally Alters the Excitatory Regulation of Spiny Projection Neurons (SPNs)

**Figure 3:**
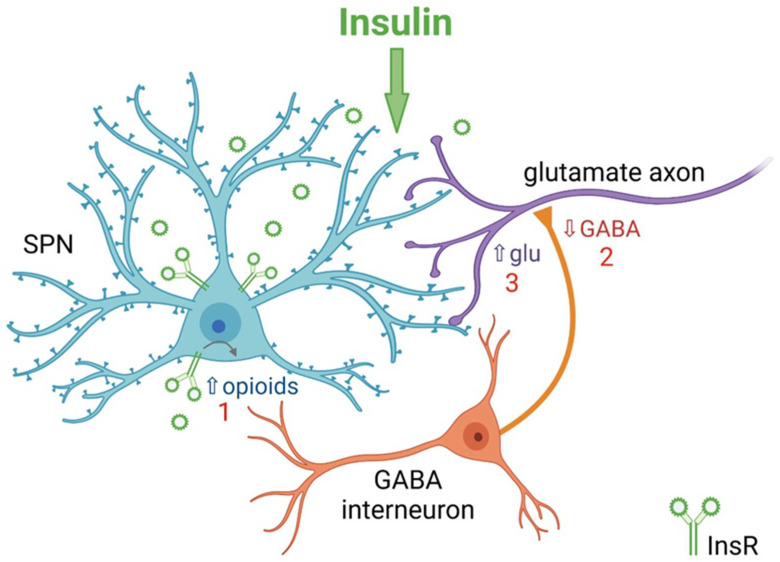
Summary of mechanism by which insulin increases excitatory regulation of striatal projection neurons (SPNs) in NAc core. 1. Activation of insulin receptors (InsRs) on GABA/opioid containing SPNs in rat nucleus accumbens (NAc) core increases the release of endogenous opioids. 2. Opioid activation of GABAergic interneurons decreases GABA release onto glutamatergic inputs from cortex and thalamus. 3. Decreased GABA release from GABAergic interneurons disinhibits glutamatergic inputs resulting in increased presynaptic glutamate (Glu) release onto dendritic spines of SPNs. Higher concentrations of insulin act at insulin-like growth factor-1 receptors (IGF-1Rs) to oppose this process (not shown). Based on [78].

In addition to its effect on ChIs and dopamine axons, insulin also acts at other striatal neurons including SPNs, thereby influencing overall striatal output. These projection neurons represent over 95% of the total neuronal population of the striatum, and can be subdivided into two classes based on expression of dopamine D1 vs. dopamine D2 receptor subtypes, as well as differential expression of endogenous opioid peptides, and different projection targets [76,77]. However, levels of InsR-mRNA appear to be similar in the two classes of SPNs (Figure 1b). Whole-cell voltage-clamp recordings of electrically evoked excitatory post-synaptic currents (eEPSCs) from individual SPNs in striatal slices show that 30 nM insulin increases the amplitude of eEPSCs via activation of InsRs on both D1- and D2-SPNs within the NAc core. By contrast, insulin concentrations ≥100 nM have the opposite effect of decreasing eEPSC amplitude through activation of IGF-1Rs [78]. Moreover, IGF-1Rs appear to provide tonic inhibition of SPNs because blocking IGF-1Rs with PPP alone increases eEPSC amplitude and also enhances the effect of 30 nM insulin. Analysis of the effect of 30 nM insulin on spontaneous miniature EPSCs (mEPSCs) show an increase in the frequency of these events, but not the amplitude, as well as a decrease in paired-pulse-ratio; all are consistent with enhanced excitatory transmission mediated by a boosting of presynaptic glutamate release. Again, a higher concentration of insulin, 100 nM, has the opposite effect through activation of IGF-1Rs, suggesting that IGF-1R-mediated reductions in excitatory transmission are also due to effects on presynaptic glutamate release [78]. Thus, insulin causes bidirectional effects on SPN excitability through activation of InsRs and IGF-1Rs. The mechanisms by which insulin modulates SPN activity appears to involve a feedback microcircuit in which insulin enhances SPN excitability, through activation of InsRs on SPNs and subsequent release of endogenous opioids, which then inhibit local GABAergic interneurons leading to disinhibition of presynaptic glutamate release that ultimately influences striatal output (Figure 3) [78].

## 6. Insulin Actions on Striatal Glial Cells

Astrocytes, like other glial cells in the brain (microglia and oligodendrocytes), play an important role in maintaining homeostasis of the brain microenvironment during all stages of life. This is achieved through maintaining the BBB, supplying trophic support to neurons, regulating local ionic concentrations, mediating synapse formation and function, pruning synapses during development and signaling across brain regions to modulate neuronal activity [79,80,81]. Moreover, astrocytes are instrumental in controlling levels of a variety of neurotransmitters/neuromodulators, including the key transmitters glutamate and GABA, as well as releasing these transmitters and other gliotransmitters [82,83,84,85].

Brain insulin has been shown to play an important role in regulating systemic glucose metabolism through astrocytic InsRs in the hypothalamus [85,86,87,88]. Moreover, insulin can act on astrocytes and other glial cells to regulate local cellular metabolism [89]. DropViz data show that striatal astrocytes express InsR mRNA (Figure 1b). Mice with astrocyte-specific InsR deletion exhibit a decrease in the peak amplitude of evoked [DA]_o_ in both dStr and NAc with no change in response duration, or in MAO levels [90]. The mechanism by which InsRs in astrocytes regulate dopamine release has been proposed to be through targeting tyrosine phosphorylation of SNARE proteins that enable exocytotic ATP release, which in turn enhances axonal dopamine release via G-protein coupled P2Y purinergic receptors (Figure 2) [35,90]. These findings add to the range of mechanisms through which insulin can alter striatal dopamine signaling.

## 7. Role of Striatal Insulin in Signaling Food Reward and Regulating Consumption

It is well established that striatal dopamine is essential for feeding [91,92]. This is clearly demonstrated by the observation that mice that are genetically incapable of synthesizing dopamine do not seek food, and will perish without intervention—even though they are physically able to consume food presented to them [93]. Although feeding behavior is essential for survival, it is complex, involving multiple brain regions orchestrating multiple interrelated components that include food-seeking behavior. These components include assessing the taste and texture (orosensory) and the nutritional value of food, which reinforce food choice, as well as knowing when to stop eating (satiation) [94,95]. An understanding of the extent to which striatal insulin influences these various aspects of feeding behavior is not yet complete; however, clues are emerging.

Functional magnetic resonance imaging (fMRI) in humans shows that intranasal insulin influences brain activity in regions that mediate reward and motivation, including the mesolimbic dopamine projection from the VTA to the NAc [29,96]. As noted in Section 2 and Section 3, insulin’s effect on dopamine release and uptake are greater in the NAc than in the dStr. The NAc is a key node of the brain reward circuitry and is fundamentally involved in reward-related learning, incentive motivation and hedonic reactivity. Energy homeostasis is achieved, in part, by peripheral signaling that modulates these behavioral functions through influence on the inputs, local circuit activity and outputs of the NAc (e.g., [36,97,98,99,100]). Certainly, InsRs and receptor mRNA have been identified in the NAc, and functional consequences of insulin exposure have been demonstrated (Figure 1a,b) [13,16,17,19,86,101]. However, the behavioral effects of NAc insulin have been investigated only recently [19,102,103,104].

### 7.1. NAc Shell Insulin Acts as a Reward Signal That Mediates Sensing of Nutrient Value

**Figure 4:**
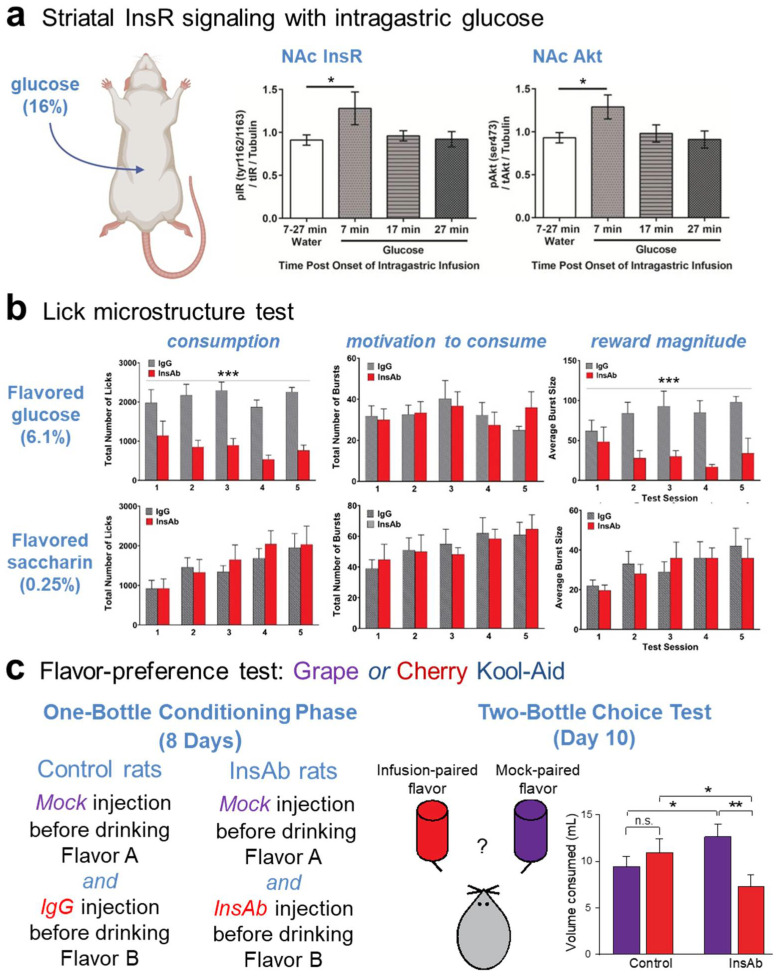
Endogenous activation InsRs in NAc shell is required for sensing nutrient value. (**a**) Phosphorylation of insulin receptors (InRs) and Akt in rat nucleus accumbens (NAc) are increased within seven minutes of intragastric glucose (16%) infusion; modified from [102]. (**b**) Lick microstructure analysis during NAc shell insulin antibody (InsAb) microinjection decreases consumption of flavored 6.1% glucose but not of non-nutritive flavored saccharin through a decrease in reward magnitude; modified from [103]. (**c**) In a two-bottle flavor preference test, InsAb rats drink less of an infusion-paired flavor vs. a mock-paired flavor than controls; modified from [19]. In (**a**–**c**), n.s. is not significant, * *p* < 0.05, ** *p* < 0.01, *** *p* < 0.001.

In the investigation of conditions under which NAc insulin signaling plays a role, it was shown that oral consumption of a 16% glucose solution by rats increases NAc InsR phosphorylation (p-Tyr1162/1163), as well as phosphorylation of the downstream signaling protein, Akt (p-ser473), within seven minutes of initiating consumption [102]. In a follow-up experiment, the same result was obtained with post-oral glucose delivery by intragastric infusion (Figure 4a) [102]. This connects insulin signaling in the NAc to the nutritive value of glucose, rather than its orosensory characteristics.

The behavioral function(s) regulated by NAc insulin signaling have been probed further using two well-established assays: lick microstructural analysis and flavor–nutrient learning. Experimental inhibition of endogenous insulin signaling in the NAc shell was achieved by local microinjection of an antibody raised against insulin. Effectiveness of the insulin antibody to bind and block local endogenous insulin was confirmed by demonstrating that its microinjection in NAc prevented oral glucose-induced InsR phosphorylation [102] and, in another assay of insulin function, blocked exogenous insulin-enhanced dopamine uptake in striatal synaptosomes [19].

Lick microstructure analysis has been in use for many years in numerous laboratories to evaluate effects of physiological and pharmacological treatments on the separate functions of hedonic impact and motivation to consume. These functions are reflected in different distinct parameters of licking behavior [105,106,107,108]. Rodent licking occurs in bursts, which are groups of licks separated by an inter-lick interval of >1 sec. The number of bursts emitted per unit time is modulated by factors that include food deprivation, gastric filling and administration of satiety-inducing agents [106,109,110,111]. Burst size is the number of licks in a burst, and is modulated by factors that include sugar concentration, contrast between alternative sugar concentrations and adulteration of sugar solution with bitter quinine [105,112,113]. Consequently, the number of bursts emitted per unit time is considered to reflect motivation, and the average number of licks per burst to reflect hedonic impact. Showing a role for insulin in the rat NAc shell, local microinjection of insulin antibody, with a non-specific IgG as the control, decreased consumption of flavored 6.1% glucose during a 30 min test session, and did so by markedly decreasing the number of licks per burst, while having no effect on the total number of bursts emitted (Figure 4b) [103]. This result indicates a decrease in glucose reward magnitude when insulin signaling is blocked by insulin antibody, with no effect on motivation to consume. Importantly, when rats were licking for flavored, non-nutritive 0.25% saccharin, insulin antibody microinjection in NAc had no effect on total consumption or any individual lick parameter (Figure 4b). Together, these data indicate that reward based on nutritive value is conveyed, at least in part, by the insulin surge that accompanies consumption, and depends on insulin signaling in the NAc shell. It was further observed that when test sessions continued beyond those in which rats received microinjections of insulin antibody, the decrease in number of licks per burst for flavored glucose persisted, but was not seen in rats that had received an equal number of insulin antibody microinjections without concurrent consumption [103]. This suggests that insulin inactivation caused subjects to associate this flavored glucose solution with low reward value.

The role of NAc insulin signaling in learning was explicitly investigated in two different flavor conditioning protocols. In the first, rats underwent a series of 30 min conditioning sessions on eight separate days. On the four odd-numbered days, one subset of rats consumed a flavored glucose solution (grape or cherry) preceded by NAc microinjection of insulin antibody. Another subset of rats received microinjections of control IgG. On the four even-numbered days, rats received mock microinjections and consumed glucose solution containing the other flavor. This was followed by a 60 min two-bottle choice test in which rats could consume unsweetened cherry or grape. Rats that had been micro-injected with insulin antibody showed a marked preference for the flavor paired with mock microinjection. Rats that had been microinjected with IgG showed equal preference for the two flavors (Figure 4c) [19]. These results provide direct evidence that insulin signaling in NAc plays a role in reinforcing flavor preference based on associated nutritive value.

In a second flavor conditioning protocol, rats again underwent a series of conditioning sessions with either grape- or cherry-flavored sweet solutions. However, one flavor was associated with 6% glucose and the other with, an initially equally preferred, 1% glucose + 0.125% saccharin; the former produced greater NAc InsR phosphorylation than the latter. In a subsequent two-bottle choice test with unsweetened flavors, rats showed a strong preference for the flavor paired with 6% glucose. If rats received microinjection of insulin antibody in the NAc shell prior to sessions with flavored 6% glucose they failed to develop preference for that solution. In contrast, if rats received microinjection of the control IgG prior to sessions with flavored 6% glucose there was no interference with acquisition of a preference for that solution [102].

This collection of behavioral results suggests that insulin’s regulation of the rewarding impact of a nutritive solution is closely related to, or homologous with, insulin-dependent reinforcement of flavor preference based on nutritive value. Encoding nutritional value is reliant on striatal dopamine signaling [114]. Moreover, both the size of lick bursts in the microstructure assay, and reinforcement of flavor–nutrient learning are known to depend on dopamine transmission via dopamine D1Rs in the NAc [115,116,117,118]. Interestingly, striatal ACh release in the NAc has been shown to increase towards the end of a feeding bout [119,120]. One would predict that insulin-regulated nAChR-dependent stimulation of dopamine release, shown in striatal slices, underlies these behavioral effects. The involvement of ACh release from ChIs and activation of nAChRs in the behavioral findings has yet to be explored.

### 7.2. NAc Core Insulin Decreases Feeding by Impairing Motivation for Consumption

As noted in Section 1, insulin’s actions in the hypothalamus play an important role in signaling satiation/satiety. In addition, the lateral hypothalamus, VTA and other brain regions and circuits have been implicated in regulating the motivational aspects of homeostatic and hedonic food intake [95,121]. For example, microinjections of insulin in the VTA can decrease hedonic feeding and preference for cues associated with food reward without decreasing effort to obtain food [34,37,38]. Recent data show that microinfusion of insulin into the NAc core decreases food intake, even in hungry rats, but unlike the result of VTA insulin infusions, NAc core insulin impairs the motivation to work for food without altering motivational responses to food cues [104]. These data, along with those showing insulin’s role in the NAc shell in reinforcing nutritive value and regulating hedonic impact without influencing motivation to consume [102,103], highlight how the relationships among insulin’s actions in the striatum, responses to food and its cues, motivation to feed, food choice, consumption and satiety are complex and nuanced. Current thinking is that this complexity arises largely from the recruitment of different microcircuits by insulin’s actions in striatal subregions acting over a range of time frames in coordination with wider neural networks.

### 7.3. Human Imaging Studies Support a Link between Striatal Insulin and Dopamine Signaling in Food Consumption

Early studies in rats showed that administration of insulin into the CSF initially increases dopamine release in the NAc but then decreases it after 30 min [122] and that activation of NAc InsRs approximately 10 min after initiation of intake, as seen in our own studies [102], corresponds to the near-peak of a meal-induced insulin surge [123]. In a more recent study in humans that combined fMRI with continuous PET imaging of dopamine receptor occupancy at 5 min intervals, Thanarajah and colleagues showed that striatal dopamine increases in two phases following ingestion of a highly palatable milkshake [124]. Each of these phases involves distinct neural circuits, with an initial increase in dopamine in the NAc immediately after intake, whereas a second delayed phase showed increased dopamine release in the dStr approximately 15 min after feeding onset. Increases in dStr dopamine have been previously correlated with meal pleasantness, as well as nutritional value of sugar [125,126]. Interestingly, individuals in the recent imaging study who had a strong dopamine release response in the first phase related to the orosensory rewarding properties of the milkshake had a weaker post-ingestion response and vice versa [124]. In another recent study using a combination of fMRI and PET imaging of dopamine receptor occupancy, tonic (basal) dopamine levels in the striatum of healthy fasted males were lower at time frames mimicking postprandial phase 15 and 30 min after intranasal insulin administration, with high responders correlating with better increases in mesocorticolimbic functional connectivity [96].

Although these findings seem at odds with the other results just discussed, it should be noted that differences likely reflect differences in the timeframe of measurements taken after food intake or intranasal administration. It has been postulated that during a feeding bout, there is an immediate increase in brain insulin. This insulin surge could activate InsRs on NAc ChIs (within 10 min) to enhance striatal dopamine release to communicate the nutritional value of the meal and shortly after enhance dopamine release in the dStr. However, insulin actions with prolonged feeding (~30 min) engage satiety systems including in the hypothalamus and VTA dopamine neurons to decrease striatal dopamine release and limit food cravings and further food intake [33,34]. Thus, insulin may act as both a reward signal and a negative-feedback signal in motivated feeding.

## 8. Dysregulated Striatal Insulin Signaling with Diet

Through actions in the striatum, hypothalamus, VTA and other brain regions, insulin can influence decisions on what, when and how much we eat. The ability of insulin to signal in the brain, however, can be altered by diet. It is well established that consuming too little or too much can have negative consequences on health; as is the case with addiction to drugs, aberrant eating patterns can develop into behavioral cycles that are difficult to break. Evidence suggests that chronic alterations in diet can have profound effects on the reward-related circuitry of the brain; brain reward thresholds are lowered by food restriction and elevated by obesity [100,127,128,129,130,131,132,133]. Increasing evidence indicates that the sensitivity of insulin signaling in the brain follows a similar pattern with chronic changes in diet.

In rodents, as in humans, circulating plasma insulin levels follow body weight [10,19,102,134]. It is well known that chronically elevated plasma insulin seen in obesity and type 2 diabetes can result in peripheral insulin resistance. Importantly, insulin resistance occurs in the brain, as well [32,134]. Studies in rodent striatum have shed light on this process through examination of InsR sensitivity and downstream signaling pathways resulting from chronic alterations in feeding state. The effect of insulin on striatal dopamine release and uptake was examined in rats fed three different diets: a food-restricted diet to lower body weight to 80% of its initial value; an energy dense high-fat-high-sugar (HF-HS) obesogenic diet with free access to a chocolate-flavored liquid (Ensure), along with chow and water; and a control ad libitum diet of chow and water [19,65,66,102]. In these studies, consequences of the obesogenic diet were examined in a pre-diabetic stage, in which glucose tolerance was unaltered.

In slices obtained from rats on the control diet, the half-maximal insulin concentration (EC_50_) for enhancing striatal dopamine release was 2–12 nM, which is within the expected nM range of insulin elevation following feeding (e.g., [2]). However, the EC_50_ for insulin-enhanced dopamine release in slices obtained from rats on the food restricted diet was ~10-fold lower, such that sub-nM insulin, which has no effect in control ad libitum fed rats, is sufficient to amplify dopamine release. This implies supersensitive InsR signaling in the brain when circulating insulin levels are chronically low. Conversely, the influence of insulin on dopamine release is lost in rats on a HF-HS diet [19], consistent with peripheral and central insulin resistance seen in human obesity [32,134]. Mirroring the influence of diet on insulin-enhanced dopamine release, the enhancing effect of insulin on dopamine uptake in striatal synaptosomes and slices is more pronounced in tissue from food restricted vs. ad libitum fed rats and is blunted in rats on a HF-HS diet [65,66].

It should be noted that InsR signaling in response to intragastric glucose is also diet sensitive. Chronic food restriction, accompanied by low basal circulating levels of insulin, increases glucose-induced InsR phosphorylation in the NAc, whereas maintenance on a HF-HS diet (chocolate Ensure), accompanied by high basal circulating levels of insulin, eliminates glucose-induced InsR phosphorylation [65]. These observations support the notion that diet-regulated levels of brain insulin set the tone for acute InsR-pathway- mediated signaling.

Is striatal insulin insensitivity with obesogenic diets a consequence of the high fat, high sugar, or both? High fat is certainly a crucial component, given that the enhancing effect of insulin on dopamine release and uptake in mouse striatal slices is also lost with a diet high in fat only [69]. Moreover, InsR-mediated signaling is disrupted in SPNs of obese male rats fed high-fat pellets, resulting in a loss of insulin’s ability to increase excitatory glutamatergic transmission in the NAc core through a decrease in InsR surface expression. At the same time, opposing influences on glutamatergic transmission mediated via IGF-1Rs are preserved [78]. Overall, these data suggest that high circulating levels of insulin (and/or other adiposity hormones, like leptin) in obesity can lead to striatal InsR insensitivity, independent of the caloric source used to induce obesity.

Dynamic diet-dependent changes in InsR signaling could have profound behavioral consequences as well. For example, food restricted and ad libitum fed rats acquire preference for an unsweetened flavor paired with 6% glucose, over 1% glucose + 0.125% saccharin, while rats maintained on the HF-HS diet that blocks glucose-induced InsR phosphorylation, do not [65]. The behavioral consequences of disabling insulin’s role in regulating the rewarding impact of a nutritive solution, with a HF-HS diet and subsequent hyperinsulinemia and InsR-subsensitivity in the NAc, has yet to be fully explored. However, it is likely that this would drive consumption of higher than ‘normal’ sugar concentrations to achieve a rewarding effect, reinforcing new food choices that are more calorically dense, and leading to increased risk or exacerbation of obesity, and eventually, type 2 diabetes. Similarly, behavioral consequences of hypoinsulinemia and supersensitive InsR signaling with FR might contribute to the aberrant eating behaviors associated with anorexia.

The conclusion of a study in human subjects that combined intranasal insulin administration, hyperinsulinemic-euglycemic clamp and fMRI was that insulin signaling in the striatum works together with that in the hypothalamus to control peripheral glucose homeostasis [135]. Paralleling peripheral insulin resistance, striatal and hypothalamic activities monitored with fMRI in obese subjects were unresponsive to insulin in this protocol. Unlike the short latency responses to insulin shown in our studies, this regulatory effect of striatal insulin exposure emerged with a latency of approximately two hours after insulin administration. Moreover, the caudate nucleus (the dStr in rodents), rather than the NAc, was the striatal subregion implicated and dopamine involvement was not established. Whether differences in the feedback to the brain from periphery, as regulated by striatal insulin sensitivity, interacts with mechanisms described for flavor–nutrient learning and hedonic reactivity is a question of interest for future research.

## 9. Dysregulated Striatal Insulin Signaling in Anxiety and Depression

Given that dopamine can influence a broad range of brain functions including mood and cognition, it is not surprising that dysfunction of striatal InsRs signaling can contribute to negative mental states. Mice with brain-wide knockout of InsRs show increasing signs of depressive-like behavior in the forced swim and tail suspension tests as they age, as well as increased signs of anxiety in other behavioral tests [75]. Implicating elevated dopamine metabolism in the mice from elevated MAO activity, treatment with antidepressant MAO inhibitors such as phenelzine, reverses depressive-like behaviors [75]. However, mice with selective deletion of InsRs in dopamine neurons show no changes in the forced swim test, elevated maze test or in sucrose preference, indicating no change in depressive or anxiety-related behavior [136]. Remarkably, mice with selective deletion of InsRs in astrocytes either from birth or in adulthood do display depressive-like and anxiety-like behaviors, including decreased sucrose preference [90]. Moreover, selective deletion of astrocytic InsRs in NAc, but not in medial prefrontal cortex, implicate the NAc in insulin-dependent mood control. As discussed in Section 6, loss of InsR signaling in astrocytes decreases ATP release from astrocytes and decreases striatal dopamine release. A causal role for impaired ATP and dopamine release in the anxiety and depressive phenotypes seen in the inducible astrocyte-specific InsR-KO mouse line was demonstrated by rescue of behavioral deficits by systemic administration of a selective P2Y ATP receptor agonist or a selective dopamine D2/D3 dopamine receptor agonist, but not a selective agonist for serotonin 5-HT1A receptors [90]. Thus, InsRs on striatal astrocytes play a key role in the regulation of mood by insulin, which could contribute to increased susceptibility to depression in insulin-resistant conditions.

## 10. Dysregulated Striatal Insulin Signaling with Age and Age-Related Disorders

InsR expression and function is known to decrease with age in multiple brain regions [137,138,139]. Thus, dysregulation of insulin signaling may contribute to age-related disorders. Given the role of the striatum and striatal dopamine in motor behavior, it is not surprising that impairment in striatal insulin signaling may influence movement-related functions, particularly in older adults in which the number of dopamine cells and striatal dopaminergic function is also diminished. Interestingly, diabetes is a risk factor for changes in gait that result in age-related falls [140,141,142,143]. Although young mice with InsRs selectively ablated in dopamine neurons show no change in locomotor activity in an open field [136], a role for InsR resistance in dopamine neurons, dopamine axons or other striatal cells in gait changes or other motor deficits with age has yet to be explored.

There is also increasing evidence that brain insulin resistance plays a role in age-related neurodegenerative disorders such as Parkinson’s disease (PD) and Alzheimer’s disease (AD) [144,145,146,147]. Indeed, epidemiological studies reveal an association between type 2 diabetes and increased risk of developing PD [147,148,149]; a movement disorder caused by an additional decline in the normal age-related progressive loss of midbrain dopamine neurons that consequently leads to depletion of dopamine in the striatum and severe motor deficits. Moreover, PD pathology and symptoms appear faster and with increased severity in this group of individuals. Animal studies also add weight to this body of evidence. In mouse models of type 2 diabetes or rats with insulin resistance induced by a high fat diet, dopamine neurons exhibit enhanced vulnerability to PD-related neurotoxins including 6-OH-DA and MPTP [150,151]. Moreover, insulin resistant mice overexpressing the PED/PEA-15 protein exhibit metabolic changes in the striatum as well as decreased striatal TH expression and dopamine content, and develop PD-like motor deficits [152]. The mechanisms involved are still being elucidated, but point to the notion that normal insulin signaling contributes to the neuroprotection of dopamine cells; activating PI3K-Akt signaling cascades via InsRs and IGF-1Rs inhibits cell death, regulates alpha-synuclein expression and aggregation, modulates autophagy and regulates mitochondrial function and inflammation [146,147]. On the other hand, PD can increase vulnerability to insulin resistance both in human patients and in animal models of PD [153]. Implicating a role for InsR dysfunction in rat striatum, InsR substrate 2 phosphorylation at serine residues, an indicator of insulin resistance, is enhanced in the 6-OH-DA model of PD [154].

Additionally, a common non-motor symptom of PD is cognitive impairment, which is reported to be higher in PD patients with diabetes than those without [155]. Moreover, insulin resistance is greater in PD patients with dementia even without diabetes [146,155]. Although impaired cognition is likely to stem from brain structures such as the hippocampus and cortex, the striatum and striatal dopamine also play an important role in cognition, and thus dysfunctional insulin signaling in the striatum could be a contributing factor [156,157]. Similarly, there is a higher risk of developing AD in those with type 2 diabetes, and conversely, higher insulin resistance in people with AD [145,158,159]. However, whether disruption of striatal insulin signaling plays a role in the symptoms or pathology of AD is unknown.

## 11. Targeting Insulin Actions in the Striatum for Therapeutic Gain

It is clear that the actions of insulin in the striatum play a significant role in ingestive behavior, both in terms of regulating food intake, and as a reward signal to develop nutrient-based food preferences. In addition, striatal insulin influences emotional and cognitive behavior which could also impact ingestive behavior and vice versa. Moreover, striatal insulin could affect motor behavior. Alteration of insulin signaling involved in mental state, as discussed in Section 9, or in aging and neurodegenerative disorders, as discussed in Section 10, would be expected to be a slow, long-term process, adding nuance to the shorter term activation of striatal neuron activity and dopamine release expected to accompany transient increases in insulin around consumption of a meal. Yet, much remains to be learned about the influence of insulin on striatal motor and motivational systems through its actions on dopamine release and uptake, neuron activity and on astrocytes. Dysregulation of each of these insulin targets could contribute to maladaptive conditions, including eating disorders, as well as in disorders involving depression, anxiety or cognitive or motor dysfunction. Thus, each target of insulin in the striatum provides a potential avenue for therapeutic intervention.

For example, loss of insulin sensitivity on DAT function in rodents on a high-fat diet is caused by impaired efficacy of downstream InsR substrates, rather than a lower number of InsRs; this InsR insensitivity can be reversed by inhibiting tyrosine phosphatase 1B, which preserves phosphorylation at tyrosine residues on InsR substrates [69]. Similarly, restoring phosphorylation of downstream Akt selectively in dopamine neurons by striatal viral injection rescues downregulation of DAT surface expression and function [160]. Thus, targeting intracellular signaling components to restore striatal InsR signaling in conditions that promote insulin resistance could be therapeutically beneficial.

Previous studies highlight the role of striatal ChIs and activation of nAChRs on dopamine axons in insulin’s ability to increase dynamic dopamine release in striatal slices [19]. This striatal microcircuit also indicates potential targets for therapeutic intervention in eating disorders. In support of this, reinforcement of flavor–nutrient learning and the size of lick bursts that provide an index of food liking are both dopamine-dependent processes, so that targeting ChIs or nAChR regulation of dopamine release could be explored. Intriguingly, it was reported recently that the adiposity hormone, leptin, also increases the excitability of ChIs and amplifies striatal dopamine release via nAChRs, albeit with the opposite efficacy among striatal subregions seen with insulin, with a greater effect in the dStr than in the NAc [161]. Leptin and insulin engage common signaling elements, including the PI3K pathway. When applied concurrently to mouse striatal slices, the effect of insulin plus leptin on evoked [DA]_o_ is not additive, suggesting the elevated leptin levels that accompany obesity might also contribute to the loss of brain InsR sensitivity in this state [161]. Further examination of leptin–insulin interactions could provide mechanistic insight into the influence of diet on the signaling of both metabolic hormones.

The potentially influential role of insulin in modulating mood and cognition may be a contributing factor in the higher rates of depression and cognitive decline seen in individuals with obesity and diabetes [31,32,162]. Conversely, individuals with depression have a higher risk of overeating and developing type 2 diabetes [163]. Thus, exploring whether treating one condition (obesity or depression) will improve the trajectory of the other is of interest [164,165,166]. For example, aerobic exercise is often recommended to combat obesity and depression although the underlying mechanisms for such benefits are largely unknown. Animal studies show that aerobic exercise increases dopamine release throughout the striatum [167] and restores deficits in dopamine release and insulin signaling via Akt in the NAc in obese rats on a high-fat diet [168]. Further studies linking the impact of exercise and insulin signaling could reveal novel signaling molecules and pathways in the brain that could be of clinical relevance. Similarly, exploiting the underlying mechanisms involved in insulin actions on striatal astrocytes in anxiety, i.e., by regulating ATP release, activation of PY2 receptors and dopamine transmission, could be explored as well [90].

Another example of how treating one condition may improve the trajectory of the other comes from the observation that there is a lower incidence of PD in those with type 2 diabetes treated with antidiabetic drugs [146,169]. Moreover, animal models of PD and individuals with PD taking antidiabetics show some protection of dopaminergic neurons and improved motor function [147]. Thus, targeting insulin signaling by repurposing current medications as well as developing new ones to manage PD progression is of current interest [147,153,170,171]. However, establishing whether striatal insulin and its striatal targets plays a role in any beneficial effects awaits future studies.

In humans, insulin is detectable in the CSF within an hour of intranasal administration [172] and can improve mood and memory and lower body weight in healthy individuals [173,174]. Given that intranasal insulin bypasses the BBB to enter the brain with negligible systemic consequences, there has been much interest in the use of intranasal insulin to treat individuals with cognitive decline, obesity/type 2 diabetes and mood disorders [174,175]. However, the involvement of the striatum in the effects of intranasal insulin actions are only just beginning to be appreciated. As mentioned in Section 7.3, fMRI studies show that intranasal insulin administration in humans does influence striatal function [29,96]. Specifically, in individuals with normal fasting insulin levels, images of food-related items (food-cues) are preferred over images of non-food items (non-food cues) and increase activity in metabolic and reward-related brain regions under control conditions. In particular, the NAc is specifically activated during food–value encoding (i.e., palatability). Intranasal insulin administration in these individuals decreases preference ratings for food cues and suppresses food-value signals by decreasing the functional strength of VTA-to-NAc connections in fMRI scans. This reveals an important role of insulin action in mesolimbic pathways for the processing of food value and salience in the human brain, which might prevent the overeating of palatable food even in an environment with an abundance of food cues. However, this is not the case in individuals with non-diabetic insulin-resistance. Under control conditions, these individuals have impaired food valuation and a lack of activation in the NAc with food cues in fMRI scans. Remarkably, however, the food-specific valuation signal in the NAc was found to increase in response to intranasal insulin and was close to those seen in the normal insulin-sensitivity group under control conditions [29]. Thus, intranasal insulin may be beneficial in modifying aberrant eating behavior in these individuals.

Intranasal delivery of insulin can also improve the motivation to move in a rat model of aging [143]. Moreover, in the 6-OHDA rodent model of PD, intranasal insulin is associated with reduced dopamine cell death as well as improved dopamine function and motor performance [176,177,178]. Given the importance of the striatum in the control of motor behavior and motivation to move, harnessing the impact of insulin signaling in the striatum might be a therapeutic approach for the treatment of impaired motor activity and cognition in normal aging, as well as in PD.

## 12. Conclusions

It is clear that insulin’s actions in the striatum are multifaceted in terms of the striatal elements involved and which insulin-sensitive receptors mediate the effects. Insulin can boost dopamine release indirectly through actions at InsRs on ChIs and astrocytes, with slower regulation by inhibiting dopamine metabolism by MAO. In addition, insulin actions on SPNs that engage local striatal microcircuits can have a direct influence on striatal and basal ganglia output. At present, information about the behavioral impact of striatal insulin signaling is limited. However, recent studies of the influence of insulin on striatal cells and circuits, as well as on striatal-mediated behaviors in rodents and humans show that insulin can influence both dopamine release and striatal output. Together, these findings show that insulin can act as a reward signal in the brain, providing a new dimension to the established roles of insulin in signaling satiety and energy homeostasis via the hypothalamus. A role for insulin as a reward signal might seem at odds with its role as a satiation/satiety signal; however, these roles are complementary, not contradictory. Indeed, insulin not only signals when to end a meal, but also to establish a memory of its nutritive value that reinforces repetition of the behavior. Other studies suggest the involvement of insulin in decreasing food intake through decreased motivation and reveal an important role of striatal insulin in maintaining mood and motor behavior. One final point is that many rodent and human studies have been conducted in males. Given that males and females can exhibit differences in feeding behavior, and are differentially susceptible to eating disorders and to anxiety-related illness, the need for future studies to examine roles of insulin in both sexes cannot be overemphasized. This is essential to provide mechanistic insight into the roles of insulin in normal brain function, as well as to lay groundwork for harnessing targets of insulin for therapeutic gain when these processes go awry.

## Data Availability

Not applicable.
